# Risk assessment tools in criminal justice and forensic psychiatry: The need for better data

**DOI:** 10.1016/j.eurpsy.2016.12.009

**Published:** 2017-05

**Authors:** T. Douglas, J. Pugh, I. Singh, J. Savulescu, S. Fazel

**Affiliations:** a Oxford Uehiro Centre for Practical Ethics, Faculty of Philosophy, University of Oxford, Suite 8, Littlegate House, St Ebbes Street, Oxford OX1 1PT, United Kingdom; b Department of Psychiatry, University of Oxford, Warneford Hospital, Oxford OX3 7JX, United Kingdom; c Oxford Health NHS Foundation Trust, Warneford Hospital, Oxford OX3 7JX, United Kingdom

**Keywords:** Violence, Forensic psychiatry, Ethics and human rights, Risk assessment, Crime prediction, Racial profiling

## Abstract

Violence risk assessment tools are increasingly used within criminal justice and forensic psychiatry, however there is little relevant, reliable and unbiased data regarding their predictive accuracy. We argue that such data are needed to (i) prevent excessive reliance on risk assessment scores, (ii) allow matching of different risk assessment tools to different contexts of application, (iii) protect against problematic forms of discrimination and stigmatisation, and (iv) ensure that contentious demographic variables are not prematurely removed from risk assessment tools.

There are currently more than 200 structured tools available for assessing risk of violence in forensic psychiatry and criminal justice [1]. These are widely deployed to inform initial sentencing, parole, and decisions regarding post-release monitoring and rehabilitation. In some jurisdictions, including Canada, New Zealand, and until 2012 in the United Kingdom, risk assessment tools are or were also used to justify indeterminate post-sentence detention. In addition, violence risk assessment tools are used to inform decisions regarding detention, discharge, and patient management in forensic and, increasingly, general psychiatry.

This article highlights some potential ethical problems posed by risk assessment tools and argues that better data on predictive accuracy are needed to mitigate these. It focuses on the use of risk assessment tools in forensic psychiatric and criminal justice settings.

## 1 Professional obligations and competing values

In the psychiatric literature, criticism of risk assessment has focused on the possibility that, in deploying risk assessment tools, mental health professionals may fail to fulfil their professional obligations to their patients [2], [3]. Health professionals are expected to make the care of their patients their first concern, to build trust, and to respect patient preferences, and this expectation is reflected in professional guidelines [4]. Some argue that the use of risk assessment tools is unjustified when it is intended to realise other values, such as justice or public protection, and does not benefit the assessed individual [5], [6], [7], [8]. Buchanan and Grounds hold that “it is inappropriate to comment on a defendant's risk unless psychiatric intervention is proposed or other benefit will result” [6]. Similarly, Mullen claims that “[r]isk assessments… are the proper concern of health professionals to the extent that they initiate remedial interventions that directly or indirectly benefit the person assessed” [8].

The use of risk assessment tools is perhaps most clearly at odds with the interests of the assessed individual where the tool is used to inform decisions regarding post-sentence detention. In this context, the default position is that the person will be released; however, if the tool indicates a high risk of violence, detention may be extended. It could be argued that deploying the tool thus runs against the individual's interest in being released as soon as possible.

In some cases, however, the application of a risk assessment tool will benefit the assessed individual. There are at least three ways in which it could confer such a benefit. First, the risk assessment may be used to identify beneficial treatments. Second, the use of a risk assessment tool may facilitate an earlier release or discharge. Suppose an individual is being considered for parole or discharge from a secure psychiatric institution, but this is likely to be refused on the basis that there is insufficient evidence for a low risk of violence. In this situation, application of a risk assessment tool may provide the evidence necessary to secure an end to detention. Third, even when a risk assessment results in further detention, it might nevertheless confer a benefit because extended detention is itself in the individual's best interests. For example, it may prevent re-offending and an even longer period of detention in the future.

Moreover, even when mental health professionals administer risk assessments that are against the assessed individual's best interests, it is not clear they thereby violate a professional obligation, for the view that medical professionals ought never to act against a patient's best interests can be contested. In the setting of infectious disease control, it would be widely accepted that physicians may sometimes compromise a patient's best interests in order to promote other values, such as the health of family members and the wider public [9], [10]. Similarly, many would hold that an obstetrician may sometimes act to protect a future child, even if this comes at some cost to the patient—that is, the prospective mother [11]. It can be argued that a parallel point holds in relation to forensic psychiatry: professionals in this field may sometimes give precedence to values besides the welfare of their own patients [12]. Those who hold that risk assessment tools should be used only when they benefit the patient may thus be overstating the ethical difficulties created by such tools.

Nevertheless, the presence of competing values in risk assessment does create a *potential* ethical problem: it is possible that some values will be unjustifiably sacrificed for the sake of others. For example, there is a risk that the interests of individual patients or prisoners will be *unjustifiably* compromised in the name of public protection, or the reverse. We will argue that a lack of high quality data on predictive accuracy compounds this ethical risk.

## 2 Predictive accuracy

Existing data suggest that most risk assessment tools have poor to moderate accuracy in most applications. Typically, more than half of individuals judged by tools as high risk are incorrectly classified—they will not go on to offend [13]. These persons may be detained unnecessarily. False positives may be especially common in minority ethnic groups [14], [15].

Rates of false negatives are usually much lower. Nevertheless, in typical cases around 9% of those classed as low risk will go on to offend [13]. These individuals may be released or discharged too early, posing excessive risk to the public. Such failures of negative prediction are frequently associated with significant controversy and outrage, as reactions to recent high profile cases demonstrate [16].

The prevalence of prediction errors does not entirely undermine the rationale for deploying risk assessment tools. To balance risk to the public against the interests of the assessed individual, some method for assessing risk is required, and risk assessment tools, even if limited in accuracy, may be the best option available. However, to mitigate the possibility of inadequate or excessive detention, the limitations of risk assessment tools need to be well understood and factored into clinical and criminal justice responses.

Unfortunately, published validation findings for the most widely used tools, which allow for predictive accuracy to be estimated in advance, frequently present a misleading picture [17]. First, though there are exceptions, most tools have not been externally validated outside of their derivation sample [18], [19]. Of particular concern, few validation studies have been conducted in women, ethnic minority populations, and individuals motivated by religious or political extremism [14], [15], [17]. Consequently, it is unclear how far reported accuracy findings can be extrapolated to new settings and populations [20]. Second, there is strong evidence that conflicts of interest are often not disclosed in this field, and some evidence of publication and authorship bias [21]. (Authorship bias occurs when research on tools tends to be published by the authors of those tools, who typically find better performance.) Third, published studies frequently present only a small number of performance measures that do not provide a full picture of predictive accuracy [22].

Thus, not only is the predictive accuracy of risk assessment tools imperfect, it is also imperfectly presented in the literature. This limited and skewed evidence base creates a risk that decision makers will rely more heavily on risk assessment scores than their accuracy warrants. To mitigate this risk, there is a need for better quality data covering more subpopulations. Validation studies should include more than just one or two performance statistics, and data on the numbers of true and false positives and negatives should be clearly presented. Conflicts of interests need to be disclosed, and reviews by authors with financial conflicts of interests should be treated with caution.

In addition to risking over-reliance on risk assessment scores, deficiencies in the evidence base also generate at least three more specific problems, which we explain below: they (i) thwart attempts to match risk assessment tools to different contexts of application, (ii) complicate efforts to determine whether risk assessment tools are unjustifiably discriminatory or stigmatising, and thereby (iii) contribute to the possibility that contentious demographic variables will be prematurely eliminated from assessment tools.

## 3 The right tool for the context

Selecting the optimal risk assessment tool for a given application requires trade-offs to be made between false negatives and false positives; attempts to reduce the number of false positives will increase the number of false negatives [23]. Tools with a low rate of false negatives (due to high sensitivity) will be most effective at protecting the public, and may garner most political support, while tools with a low rate of false positives (due to high specificity) will best protect the rights and interests of prisoners and psychiatric patients.

The optimal balance between false positives and false negatives is an ethical issue and will depend on the social and political context in which the tool is to be used [24]. For example, avoidance of false positives may be more important in jurisdictions with less humane detention practices than in jurisdictions with more humane practices, since the less humane the conditions of detention, the greater the harm false positives will tend to impose on the assessed individual [25].

The appropriate balance between false positives and false negatives will also depend on the stage in the criminal justice process or patient pathway at which the tool will be deployed. For instance, suppose that a risk assessment tool is used to inform decisions about post-sentence detention in a setting where an individual's initial sentence is proportionate to their degree of responsibility and the seriousness of the crime. Detaining the individual beyond the end of the initial sentence thus involves imposing a disproportionately long period of detention. In this context, special care should be taken to avoid false positives, and there may be grounds to prefer a tool with a very low false positive rate to one that is overall more accurate.

However, the situation is different when a tool is used to inform parole decisions. In this context, false positives may lead to refusal of parole and an unnecessarily long period of incarceration from the point of view of public protection. Yet if we assume that the initial sentences are themselves proportionate, then the overall period of detention for ‘false positive’ individuals will remain within the upper limit set by considerations of proportionality. In this context it may be more important to avoid false negatives.

Matching risk assessment tools to different contexts of application thus requires trade-offs between positive and negative predictive accuracy. For each context, we must first decide which type of accuracy to prioritise to which degree, and then select a tool that reflects this priority. Unfortunately, in the absence of reliable data, it is not possible to make the latter decision confidently. There is a need for studies using representative samples for relevant subpopulations, avoiding highly selected samples, and presenting performance measures that allow false negative and false positive rates to be reliably estimated for a particular application.

## 4 Discrimination and stigmatisation

Some argue that singling out individuals for unfavourable treatment on the basis of their demographic characteristics amounts to unjustified discrimination. This criticism is often levelled at racial profiling by police and airport security [26]. A similar concern might be raised regarding risk assessment tools that take into account an individual's demographic characteristics such as ethnicity, age, immigration status and gender. It has been suggested that risk assessment tools should employ only ‘individualised’ information, such as information about declared plans and desires based on face to face interviews [17], [27], though, even then, judgments may be subject to implicit biases based on the demographic characteristics of the individual being assessed [28].

However, the requirement to utilise only individualised information is overly restrictive. Many would argue that demographic profiling is discriminatory, or problematically so, only when the demographic variables used are recognised social groups (such as ethnic or gender groups) [29], or certain kinds of recognised social groups, for instance, those whose membership is unchosen [30], or that have historically been subject to oppression [31]. Risk assessment tools could theoretically exclude such variables.

In reply, it might be argued that exclusion of such variables is insufficient to avoid moral concerns. First, even if the problematic demographic variables are formally excluded from the analysis, they may continue to exert an influence; there remains the potential for implicit bias in the application of risk assessment tools and interpretation of risk scores [14], [15], [17]. Second, even if the problematic demographic variables are formally excluded from the analysis and there is no implicit bias in applying the tools, there may still be a correlation between membership of certain demographic groups and risk score. For example, members of a particular ethnic group may be more likely than average to receive high risk scores. Some may hold that such a correlation is problematic, especially if it is due to past wrongdoing against members of the demographic group in question (e.g., members of the ethnic group are indeed more likely to offend, but only because they are victims of unjust social exclusion), if the correlation does not reflect a true difference in risk (e.g., false positives occur more frequently than average in the minority ethnic group), or if the correlation is likely to lead to stigmatisation of the group deemed to be higher risk.

However, even if the use of risk assessment tools does involve a problematic form of discrimination or stigmatisation, it could nevertheless be justified if the case in favour of using the information is powerful enough. The parallel with racial profiling in airport screening is instructive here. Airport screening is a limited resource and there are reasons to deploy it to detect the maximum number of would-be terrorists. If profiling enables a far greater number of terrorist attacks to be prevented with the resources available than any other policy, and if the cost to those profiled is low, then it is arguably justified even if somewhat problematic, for example, because discriminatory or stigmatising. Similarly, the resources available for the prevention of violence are limited, and if deploying a risk assessment tool prevents far more violence than could otherwise be prevented with the resources available, it might be justified even if it does raise some concerns about discrimination and stigmatisation.

Nevertheless, it is important that risk assessment tools deploy the most specific predictive information available. Arguably, what is most objectionable about some forms of racial profiling is that they deploy racial appearance as a predictor when more specific predictors of security threat are available and, were these predictors used, racial appearance would add no further predictive value [32], [33]. In such circumstances, use of racial appearance seems *unnecessary*.

Similarly, it may be problematic to use demographic predictors in risk assessment tools when more specific predictors of future offending are available and these predictors would render the use of demographic categories redundant.

Unfortunately, the lack of good evidence on accuracy makes it difficult to ascertain whether existing tools do use the most specific predictors available. To determine this, we would need to be able to compare the accuracy of more specific and less specific tools using relevant, reliable and unbiased data on accuracy. Currently deployed tools frequently do use demographic factors such as age and immigration status as predictors, and although recent evidence suggests that including such demographic factors improves predictive accuracy [34], [35], further data are needed to confirm this.

In the absence of these data, there are two risks. On the one hand, mental health professionals may continue to employ coarse demographic variables that result in unnecessary discrimination or stigmatisation. On the other, given growing public concern regarding the use of such variables [36], [37], professionals or policy makers may prematurely remove them from risk assessment tools [38]. Before variables are removed because they are potentially contentious, high quality research that uses transparent methods and presents all relevant outcomes should investigate whether the demographic factors included in current tools add incremental validity to tool performance [34].

## Funding

This work was supported by grants from the Wellcome Trust (100705/Z/12/Z, WT086041/Z/08/Z, #095806, WT104848/Z/14/Z), and the Uehiro Foundation on Ethics and Education.

## Disclosure of interest

SF has published research on risk assessment, including as part of a team that has derived and validated one tool for prisoners with psychiatric disorders.
